# Evaluating sarcopenia prevalence and SARC-F effectiveness in elderly Spanish women with RA: a comparative study of EWGSOP criteria

**DOI:** 10.3389/fmed.2024.1392604

**Published:** 2024-05-10

**Authors:** Lidia Valencia-Muntalà, Carmen Gómez-Vaquero, Maribel Mora, Laura Berbel-Arcobé, Diego Benavent, Javier Narváez, Xavier Juanola, Joan M. Nolla

**Affiliations:** Department of Rheumatology, Bellvitge Biomedical Research Institute (IDIBELL)-Hospital Universitari de Bellvitge, University of Barcelona, Barcelona, Spain

**Keywords:** sarcopenia, EWGSOP criteria, rheumatoid arthritis, elderly patients, SARC-F questionnaire, prevalence, muscle strength, diagnostic criteria

## Abstract

**Introduction:**

The European Working Group on Sarcopenia in Older People (EWGSOP) has put forward two key proposals for diagnosing sarcopenia: the EWGSOP1 in 2010 and the EWGSOP2 in 2019. These proposals are currently the most widely used guidelines for diagnosing sarcopenia. However, data on the prevalence of sarcopenia in patients with rheumatoid arthritis (RA) based on EWGSOP criteria are limited. This study aimed to: (a) establish the prevalence of sarcopenia in an elderly Spanish cohort of women with RA using both EWGSOP1 and EWGSOP2 criteria; and (b) evaluate the effectiveness of the SARC-F questionnaire in detecting sarcopenia.

**Methods:**

In this observational, cross-sectional study, 67 women aged over 65 years who met the ACR 2010 criteria for RA were consecutively recruited from a tertiary university hospital. Assessments included: (a) demographic and anthropometric data; (b) RA-related variables (disease history, analytical evaluation, activity, disability, quality of life); and (c) sarcopenia-related variables (muscle strength, gait speed, skeletal muscle mass, and SARC-F questionnaire). The prevalence of sarcopenia was determined using both EWGSOP1 and EWGSOP2 criteria. Furthermore, the effectiveness of the SARC-F questionnaire for detecting sarcopenia were calculated.

**Results:**

The prevalence of sarcopenia was 43% according to the EWGSOP1 criteria and 16% according to the EWGSOP2 criteria. Patients diagnosed with sarcopenia based on the latter criteria also met the EWGSOP1's criteria for sarcopenia. Agreement between the two sets of EWGSOP criteria was poor. The SARC-F questionnaire demonstrated an inherently high sensitivity (100%) as well as good specificity (75%) and diagnostic accuracy (79%) in detecting sarcopenia according to EWGSOP2 criteria.

**Conclusions:**

The prevalence rate of sarcopenia among elderly Spanish women with RA varies significantly depending on whether EWGSOP1 or EWGSOP2 criteria are applied. The SARC-F questionnaire is effective for predicting sarcopenia when used in conjunction with the EWGSOP2 criteria, which is currently the most accepted standard in clinical practice.

## Introduction

Sarcopenia, a progressive and generalized skeletal muscle disorder characterized by the accelerated loss of muscle mass and function, is associated with increased adverse outcomes such as falls, functional decline, frailty, and mortality (1).

At present, there is no universally accepted operational definition of sarcopenia. However, the proposals published by the European Working Group on Sarcopenia in Older People (EWGSOP), first in 2010 (EWGSOP-1) (2) and subsequently in 2019 (EWGSOP-2) (3), remain the predominant criteria in use. These frameworks advocate for a sequential diagnostic strategy, despite employing different criteria (4). Specifically, EWGSOP-1 defines sarcopenia through the concurrent observation of low muscle mass and diminished muscle function, indicated by reduced muscle strength or compromised physical performance. In contrast, EWGSOP-2 defines sarcopenia by combining low muscle mass and strength, using physical performance evaluations to classify the severity of the condition. Furthermore, EWGSOP-2 recommends use of the SARC-F questionnaire (5) as a tool to identify individuals likely suffering from sarcopenia.

The diagnostic concordance between these two methodologies is recognized as minimal, which has led to disparities in the reported prevalence of sarcopenia, affecting both the general population (6) and individuals with specific conditions (7, 8).

Rheumatoid arthritis (RA), the most commonly diagnosed systemic autoimmune disease, is a complex rheumatic condition characterized by persistent, progressive articular and extra-articular manifestations, ultimately contributing to heightened disability and mortality rates (9, 10).

It is acknowledged that individuals with RA are at an elevated risk for developing sarcopenia compared to the general population (11). Nevertheless, the reported prevalence of sarcopenia among RA patients is highly variable, contingent on the diagnostic definition applied and the demographics of the study population (12).

This study aims to determine the prevalence of sarcopenia within a cohort of elderly Spanish women with RA, examining the application of both EWGSOP-1 and EWGSOP-2 diagnostic criteria. While EWGSOP-2 is the prevailing standard, comparing the two strategies may provide insights into the evolution of diagnostic practices and their potential implications for patient care. Additionally, this research seeks to evaluate the effectiveness of the SARC-F questionnaire in detecting sarcopenia within this demographic.

## Materials and methods

### Study population

This observational, cross-sectional study recruited women aged over 65 who met the ACR 2010 criteria for RA, as established during routine visits to the rheumatology service of a tertiary university hospital. We excluded patients with diseases that could significantly affect their condition, such as neoplasms, cardiac or respiratory insufficiency, and chronic liver or kidney disease.

All participants provided written consent, and the study received approval from the local ethics committee.

### Study variables

#### Demographic and anthropometric data

- Age.
- Body mass index (BMI). BMI is the ratio of human body weight to squared height expressed in kg/m^2^. It has been categorized as follows: < 18.5 kg/m^2^ is considered underweight; from 18.5 to 25 kg/m^2^, normal range; from 25 to 30 mg/m^2^, overweight; and >30 kg/m^2^, obese.

#### RA assessment

- Evaluation of RA history: (a) disease duration; (b) current treatment (glucocorticoids, conventional disease-modifying antirheumatic drugs, biological disease-modifying antirheumatic drugs, Jak inhibitors); (c) rheumatoid factor seropositivity; and (d) positivity of anti-citrullinated peptides antibodies (ACPA).
- Analytical evaluation. We considered the following parameters: (a) erythrocyte sedimentation rate (ESR); (b) C-reactive protein (CRP); and (c) hemoglobin levels. The values corresponding to the last analytical study carried out were considered.
- Evaluation of RA activity. We used two indices: (a) the Disease Activity Score 28 (DAS28) and the Routine Assessment of Patient Index Data 3 (RAPID3).
  a) DAS28 (13) is a composite index of disease activity comprising tender and swollen joint counts in 28 joints, the Patient's Global Assessment of Disease Activity and the ESR. The higher the score, the higher the activity level. A value < 2.6 suggests disease remission, a value between ≥2.6– ≤ 3.2 suggests low disease activity, a value >3.2– ≤ 5.1 suggests moderate disease activity and, finally, a value >5.1 suggests high disease activity.
  b) RAPID3 (14) is a validated index for measuring disease activity in patients with RA that includes three measures self-reported by the patient: pain, physical function, and global assessment of the disease. The higher the score, the higher the activity level. A value ≤ 3 suggests disease remission, a value between 3.01–6 suggests low disease activity, a value between 6.01–12 suggests moderate disease activity and a value >12 suggests high disease activity.
- Evaluation of disability. We used the Health Assessment Questionnaire (HAQ) (15). This questionnaire assesses physical functioning as difficulty performing daily living activities; the score ranges from 0 to 3. The higher the score, the higher the disability level.
- Evaluation of health-related quality-of-life. We used the SF-12 questionnaire (16), which consists of 12 questions that measure 8 health domains to assess physical and mental health. Physical health-related domains include general health, physical functioning, physical role, and body pain. Mental health-related scales include vitality, social functioning, emotional role, and mental health. For each participant, we then calculated two summary scores using the SF-12—physical and mental health—utilizing the weighted means of the eight domains.

### Sarcopenia assessment

Sarcopenia was assessed by two different methods: EWGSOP-1 and EWGSOP-2 criteria.

Muscle strength was evaluated with a calibrated handheld Jamar type dynamometer (Kern hand grip digital dynamometer 80K1). Two trials for each hand were performed and the best result from the strongest hand was used. The cutoffs points considered were < 20 kg for the EWGSOP-1 criteria and < 16 kg for the EWGSOP-2 criteria.

Gait speed, measured in meters/second (m/s), was evaluated by the 6-m gait test, where the participant walked along a straight 6-meter track and the time was measured with a stopwatch. The cutoffs points considered were < 0.8 m/s, both in the EWGSOP-1 and EWGSOP-2 criteria.

Muscle mass was assessed by calculating the Skeletal Mass Index (SMI). SMI is established by the following formula: appendicular skeletal muscle mass/height^2^. The examinations were made with a densitometer Hologic Horizon W (Hologic Inc., Bedford, MA), recording fat and lean mass in the arms, trunk, and legs. The patient is placed supine, centered on the table with arms stretched to the sides of the body, hands facing the legs without touching them and the thumbs upwards. The cutoff point in both criteria is a value ≤ 5.67 kg/m^2^.

As required by the EWGSOP-2 criteria, the SARC-F (5) was applied as a screening tool for sarcopenia. It includes five components: strength, assistance walking, rising from a chair, climbing stairs, and history of falls. The score ranges from 0 to 10. Cutoff value of ≥4 suggest the presence of sarcopenia and indicate the need for further evaluation.

According to the EWGSOP-1 criteria, sarcopenia is considered when a patient presents low muscle mass with low muscle strength or poor physical performance. According to the EWGSOP-2 criteria, sarcopenia is considered possible when a patient presents low muscle strength and low muscle mass. Sarcopenia is considered severe, according to the EWGSOP-1 criteria, when a patient presents any anomaly in the three components analyzed (low muscle mass, low muscle strength, and poor physical performance). Sarcopenia is considered severe, according to the EWGSOP-2 criteria, when a patient with sarcopenia additionally presents poor physical performance.

EWGSOP-2 includes the category of “probable sarcopenia” when a patient presents low muscle strength with normal muscle mass.

### Statistical analysis

We calculated the necessary sample size based on a 4.5% expected prevalence of sarcopenia in RA (12), using the formula: *n* = *Z*^2^ × *p* × (1–p)/*E*^2^. Here, *Z* is the Z-score for a 95% confidence level (1.96), *p* is the prevalence rate (0.045), and *E* is the margin of error (5%). The calculation yielded a sample size of 67 participants.

Data are presented as the mean plus or minus the standard deviation for continuous variables and as a number and percentage for categorical variables. Prevalence rates are given as percentages. Continuous variables were tested for normality using the Kolmogorov-Smirnov test. Differences among parametric variables were assessed using ANOVA; for non-parametric variables, we used the U-Mann-Whitney or Kruskal-Wallis tests, when indicated. Differences among categorical variables were evaluated by the chi-squared test.

We assessed the level of agreement between the classifications of sarcopenia according to the two different criteria through Cohen's kappa coefficient. Interpretations based on kappa values are as follows: < 0: less agreement than would be expected by chance; 0 ≤ *k* ≤ 0.2: slight agreement; 0.21 ≤ *k* ≤ 0.4: fair agreement; 0.41 ≤ *k* ≤ 0.6: moderate agreement; 0.61 ≤ *k* ≤ 0.8: substantial agreement; 0.81 ≤ *k* ≤ 1: almost perfect agreement.

We also conducted a comprehensive evaluation of the effectiveness and precision of SARC-F. Sensitivity, specificity, positive predictive value (PPV), negative predictive value (NPV), and diagnostic accuracy were assessed. Sensitivity measures the proportion of true positive cases correctly identified by the test among all actual positive cases. Specificity assesses the test's ability to correctly identify negative cases among those without the studied condition. Positive Predictive Value (PPV) represents the probability that a positive test result is true. Negative Predictive Value (NPV) indicates the probability that a negative test result is true. Diagnostic accuracy reflects the proportion of true results, both positive and negative, in relation to the total results obtained.

## Results

Our study included a total of 67 patients, as detailed in Table 1. According to the EWGSOP-1 criteria, 43.3% (29/67) of the patients were diagnosed with sarcopenia, and 7.5% (5/67) exhibited severe sarcopenia. Using the EWGSOP-2 criteria, the prevalence of sarcopenia was found to be lower at 16.4% (11/67), with severe sarcopenia observed in 6% (4/67) of the patients. Additionally, 26.9% (18/67) were classified with probable sarcopenia under EWGSOP-2 criteria.

**Table 1:** Patient characteristics in accordance with the presence of sarcopenia based on the EWGSOP1 and EWGSOP2 criteria.

		**EWGSOP1**			**EWGSOP2**		
	**All patients (n: 67)**	**Without sarcopenia (n: 38)**	**With sarcopenia (n: 29)**	***p***	**Without sarcopenia (n: 56)**	**With sarcopenia (n: 11)**	***p***
Age (years)	72.6 ± 6.2	71.8 ± 5.0	73.7 ± 7.4	ns	72.6 ± 6.2	72.7 ± 6.2	ns
BMI (kg/m^2^)	27.4 ± 4.8	29.6 ± 4.9	24.6 ± 2.9	< 0.001	27.7 ± 5.1	26.1 ± 2.7	ns
Underweight (*n*, %)	0	0	0		0	0	
Normal range (*n*, %)	23 (34.3%)	8 (21.1%)	15 (51.7%)		19 (33.9%)	4 (36.4%)	
Overweight (*n*, %)	25 (37.3%)	11 (28.9%)	14 (48.3%)	< 0.001	18 (32.1%)	7 (63.6%)	< 0.05
Obese (*n*, %)	19 (28.4%)	19 (50%)	0		19 (34%)	0	
Disease duration (years)	17.9 ± 9.8	15.7 ± 9.8	20.6 ± 9.3	< 0.05	16.8 ± 9.6	23.3 ± 9.9	< 0.05
RF seropositivity (*n*, %)	40/57 (70.2%)	21/32 (65.6%)	19/25 (76%)	ns	32/48 (66.6%)	8/9 (88.8%)	ns
ACPA positive (*n*, %)	44/59 (74.6%)	26/34 (76.5%)	18/25 (72%)	ns	35/50 (70%)	9/9 (100%)	ns
ESR (mm/h)	22.6 ± 16.5	20.5 ± 14.4	25.3 ± 18.7	ns	21.6 ± 15.6	22.6 ± 16.5	ns
CRP (mg/dL)	4.9 ± 7.3	4.4 ± 6.7	5.6 ± 8	ns	4.3 ± 6	8.0 ± 12	ns
Hemoglobin (g/dL)	13.5 ± 10.2	13.8 ± 10.2	13.1 ± 8.8	< 0.01	13.6 ± 9.8	12.8 ± 10	< 0.05
DAS28	2.8 ± 1.0	2.6 ± 1	3 ± 0.98	ns	2.7 ± 1	3 ± 0.9	ns
Remission (*n*, %)	29 (43.3%)	20 (52.6%)	9 (31%)		26 (46.4%)	3 (27.3%)	
Low disease activity (*n*,%)	21 (31.3%)	10 (26,3)	11 (37,9)		16 (28.6%)	5 (46.4%)	
Moderate disease activity (*n*,%)	16 (23.9%)	8 (21.1%)	8 (27.6%)	ns	13 (23.2%)	3 (27.3%)	ns
High disease activity (*n*, %)	1 (1.5%)	0	1 (3.5%)		1/56 (1.8%)	0	
RAPID3	9.6 ± 7.5	9.4 ± 7.8	9.7 ± 7.2	ns	8.6 ± 7.2	14.3 ± 7.2	< 0.05
Remission (*n*, %)	20/64 (31.2%)	12/35 (34.3%)	8 (27.6%)		18/53(34%)	2 (18.2%)	
Low disease activity (*n*, %)	3/64 (4.7%)	1/35 (2.9%)	2 (6.9%)		3/53 (5.6%)	0	
Moderate disease activity (*n*, %)	19/64 (29.7%)	11/35 (31.4%)	8 (27.6%)	ns	18/53 (33.9%)	1 (9.1%)	< 0.05
High disease activity (*n*, %)	22/64 (34.4%)	11/35 (31.4%)	11 (37.9%)		14/53 (26.5%)	8 (73.7%)	
HAQ	0.15 ± 0.34	0.10 ± 0.16	0.22 ± 0.48	ns	0.12 ± 0.32	0.29 ± 0.42	ns
**SF-12**							
Mental health	44.7 ± 11.4	46.4 ± 10.3	42.4 ± 12.6	ns	45.0 ± 11.2	43.6 ± 13.0	ns
Physical health	37.6 ± 9.3	37.6 ± 9.5	37.6 ± 9.1	ns	38.7 ± 9.2	30.9 ± 7.2	< 0.05
**Current medication**							
Glucocorticoids (n,%)	31 (46.3%)	17 (44.7%)	14 (48.3%)		24 (42.8%)	7 (63.6%)	
cDMARDs (n,%)	56 (83.6%)	36 (94.7%)	23 (79.3%)		50 (89.3%)	9 (81.8%)	
bDMARDs (n, %)	27 (40.3%)	12 (31.6%)	15 (51.7%)	ns	22 (39.3%)	5 (45.5%)	ns
Jak inhibitors (n,%)	1 (1.5%)	0	1 (3.5%)		1(1.8%)	0	
SMI	5.48 ± 0.79	5.86 ± 0.64	4.97 ± 0.69	< 0.001	5.61 ± 0.69	4.76 ± 0.93	< 0.01
SMI ≤ 5.67 Kg/m^2^ (*n*, %)	40 (59.7%)	11 (28.9%)	29 (100%)	< 0.001	29 (51.8%)	11 (100%)	< 0.01
Grip strength < 16 Kg (*n*, %)	40 (59.7%)	15 (39.5%)	25 (86,2%)	< 0,001	29 (51.8%)	11 (100%)	< 0.01
Gait speed < 0.8 m/s (*n*, %)	53 (79.1%)	12 (31.6%)	9 (31%)	ns	17 (30.4%)	4 (36.4%)	ns

BMI, body mass index; RF, rheumatoid factor; ACPA, anti-citrullinated peptides antibodies; ESR, erythrocyte sedimentation rate; CRP, C-reactive protein; DAS28, Disease Activity Score 28; RAPID3, Routine Assessment of Patient Index Data 3; HAQ, Health Assessment Questionnaire; cDMARDs, conventional disease-modifying antirheumatic drugs; bDMARDs; biological disease-modifying antirheumatic drugs; SMI, Skeletal Mass Index. ns, not significant.

All patients diagnosed with sarcopenia under EWGSOP-2 criteria also met the EWGSOP-1 criteria for sarcopenia. However, the agreement between the two sets of criteria was poor, as indicated by a Cohen's kappa coefficient of 0.409 (*p* < 0.001), demonstrating significant discrepancies in how each set of criteria categorizes sarcopenia.

The mean SARC-F questionnaire score among the cohort was 2.9 ± 1.9, with 62.7% (42/67) of patients scoring 4 or higher. Patients identified with sarcopenia by EWGSOP-2 criteria had a significantly higher mean SARC-F score of 5.1 ± 1.5, compared to 2.5 ± 1.6 for those without sarcopenia (*p* < 0.001).

The diagnostic performance of the SARC-F questionnaire is detailed in Table 2, showing its sensitivity, specificity, predictive values, and diagnostic accuracy for diagnosing sarcopenia using EWGSOP-2 criteria.

**Table 2:** Sensitivity and specificity of SARC-F with predictive values and diagnostic accuracy.

	**Sensitivity**	**Specificity**	**PV+**	**PV–**	**Diagnostic accuracy**
Sarcopenia by EWGSOP-2	100%	75%	44%	100%	79%
Low grip strength	45%	74%	72%	48%	57%
Low SMI	35%	59%	56%	38%	45%
Slow gait speed	57%	72%	48%	79%	67%

PV+, Positive Predictive Value; PV–, Negative Predictive Value.

## Discussion

Recent advancements in RA treatment have markedly improved patient outcomes (17), enabling significant reductions in clinical symptoms, and even disease remission, by targeting inflammatory signals. Alongside the progress being made in managing RA activity, there's a growing recognition of associated comorbidities. Beyond traditional ones, conditions such as sarcopenia are now acknowledged as important considerations in clinical practice (18).

Sarcopenia, a condition marked by muscle loss, significantly impacts elderly patients by increasing the incidence of falls and hospitalization risks, reducing daily living activity capabilities, and elevating morbidity and mortality rates. Its prevalence among older populations notably contributes to frailty and disability, presenting substantial social and economic challenges (19).

Sarcopenia is traditionally classified into one of two categories: primary, attributed solely to aging; and secondary, stemming from other conditions like diseases or treatments. Though considered somewhat outdated, this classification system not only highlights sarcopenia's potential role as a symptom of underlying diseases (1), including cancer, liver, heart, endocrine, or kidney disorders, but also emphasizes its importance as a comorbidity.

RA-induced joint inflammation leads to pain, joint destruction, and reduced physical activity (10). Given that reduced physical activity and chronic inflammation are sarcopenia risk factors, assessing its prevalence in RA patients is pertinent.

Currently, sarcopenia is often undiagnosed, and while universal screening is impractical, a case-finding strategy (1) for opportunistic detection is recommended. This approach is crucial in patients with at higher risk of sarcopenia, such as in older adults or those with chronic diseases, as it will help identify and manage this condition more effectively.

It is well established (20) that the prevalence of sarcopenia in free-living older adults is lower when diagnosed according to EWGSOP-2 criteria vs. EWGSOP-1 criteria. To date, only three studies have compared these criteria in patients with specific diseases. Almeida et al. (7) analyzed both sets of criteria in 57 patients with non-alcoholic fatty liver disease (mean age: 52.7 ± 11.3 years; 75.4% females), finding sarcopenia in 3.5% of cases using only the EWGSOP-1 criteria. Valent et al. (8), studying 81 Parkinson's disease patients (mean age: 73.8 ± 5.3 years; 45% females), reported sarcopenia prevalences of 51.9% with EWGSOP-1 and 28.4% with EWGSOP-2. Lastly, de Freitas et al. (21) examined 242 patients with type 2 diabetes mellitus (mean age: 68.3 ± 5.6 years; 54% females), observing sarcopenia prevalences of 16.9% with EWGSOP-1 and 7% with EWGSOP-2.

We have established that, in the context of RA, the prevalence of sarcopenia significantly diverges depending on whether EWGSOP1 or EWGSOP2 criteria are applied, highlighting a marked discrepancy in agreement between these two diagnostic approaches. The question of whether this variance stems from an overestimation by EWGSOP1 or an underestimation by EWGSOP2 remains to be elucidated through longitudinal studies. Importantly, it must be noted that EWGSOP1 (2) prioritizes muscle mass as the principal diagnostic criterion, whereas EWGSOP2 (3) shifts the focus toward low muscle strength as the primary diagnostic measure. Evidence suggests that muscle strength is a more reliable predictor of adverse health outcomes commonly associated with sarcopenia, including diminished quality of life, increased disability, and higher mortality rates in older populations residing in the community (22). This distinction underscores the need for a nuanced understanding of sarcopenia's diagnostic criteria and their implications for patient outcomes.

Furthermore, gender-specific factors and the severity of RA could have also played critical roles in influencing these prevalence rates. Women with RA often exhibit different clinical outcomes compared to men, which might be reflected in their sarcopenic status when assessed under varying diagnostic criteria. Moreover, the systemic inflammation associated with RA and its treatments can variably affect muscle strength and mass, potentially exacerbating sarcopenia under different diagnostic frameworks.

The data observed in this study has revealed a starker contrast between the two diagnostic tools than what has been reported in prior studies (7, 8, 21). Specifically, the prevalence of sarcopenia identified using EWGSOP-2 criteria was nearly one-third of that detected with EWGSOP-1 criteria. This pronounced difference may be attributed not only to potential disease-specific variations, but also to methodological differences in our approach compared to other studies. Unlike previous research that utilized Bioelectrical Impedance Analysis (BIA) for muscle mass assessment, our study employed Dual-Energy X-ray Absorptiometry (DXA), the gold standard technique for analyzing muscle mass. This methodological distinction likely contributes to the observed discrepancy in sarcopenia prevalence between the two sets of diagnostic criteria.

Information regarding the prevalence of sarcopenia in RA patients, as defined by any of the EWGSOP strategies, remains limited. Specifically, there is a lack of data concerning the frequency of this condition when applying the EWGSOP-1 criteria. Table 3 compiles the primary outcomes from studies published to date (12, 23, 24) that have assessed the prevalence of sarcopenia using the EWGSOP-2 criteria. The prevalence observed has been very close to that reported by Cano-Garcia et al. (24) among a cohort of 77 patients whose clinical and demographic characteristics closely align with those of the current series.

**Table 3:** Patient characteristics and prevalence of sarcopenia by EWGSOP-2 criteria in RA studies.

	**Brance et al. (23)**	**Dietzel et al. (12)**	**Cano-Garcia et al. (24)**	**Present study**
Number of patients	105	289	76	67
Age	53.3 ± 13.4	59.4 ± 11.3	71.0 ± 4.8	72.6 ± 6.2
Female sex	82.9%	80%	78.9%	100%
BMI	26.96 (23.4–29.9)	27.0 ± 4.5	28.1 ± 1.0	27.4 ± 4.8
Disease duration	6.0 (2.5–14)	9 (12)^*^	18 ± 7.8	17.9 ± 9.8
RF seropositivity	80.4%	79%	75%	70.2%
ACPA positive	ND	87%	72%	74.6%
DAS28	3.6 (2.8–5.0)	2.1 (1.3)^*^	2.9 ± 1.1	2.8 ± 1.0
HAQ	ND	0.5 (0–1.3)^*^	1.28 ± 0.79	0.15 ± 0.34
Glucocorticoids	62.9%	53%	57.9%	46.3%
cDMARDs	71.4%	68%	59.2%	83.6%
bDMARDs	28.6%	46%	73.7%	40.3%
Jak inhibitors	0	8%	0	1.5%
Prevalence of probable sarcopenia	ND	24.6%	46.1%	26.9%
Prevalence of confirmed sarcopenia	19%	4.5%	15.8%	16.4%
Prevalence of severe sarcopenia	6.9%	0	1.3%	6%

^*^The data are presented as the median (Interquartile Range, IQR). BMI, body mass index; RF, rheumatoid factor; ACPA, anti-citrullinated peptides antibodies; DAS28, Disease Activity Score 28; HAQ, Health Assessment Questionnaire; cDMARDs, conventional disease-modifying antirheumatic drugs; bDMARDs; biological disease-modifying antirheumatic drugs; ND: not done.

As anticipated, individuals diagnosed with sarcopenia according to both criteria exhibited lower SMI and grip strength. However, it was surprising to note that there were no significant differences in age or gait speed between the groups. Furthermore, while no marked differences were evident in most RA-specific variables, exceptions were noted in terms of disease duration and hemoglobin levels.

Our findings indicate the SARC-F screening questionnaire offers an inherently high sensitivity (100%), a sine qua non condition for diagnosis, as well as good specificity (75%) and diagnostic accuracy (79%) for identifying sarcopenia in RA patients when used alongside the EWGSOP-2 criteria. However, SARC-F is not a good indicator of altered grip strength, SMI or gait speed. Then, in our cohort, the predictive power or SARC-F for decreased muscle strength, mass, and function is notably low. It is conceivable that structural damage associated with RA may introduce a confounding variable, potentially impacting its predictive accuracy.

In healthcare practice, the SARC-F questionnaire seems useful as a screening tool for the presence of sarcopenia in elderly female patients with RA. From an operational perspective, its administration combined with the determination of muscle strength, would help to identify patients with RA who are at risk of developing sarcopenia, eligible to start a multidisciplinary program to prevent it.

The present study has several limitations: (a) its cross-sectional design precludes establishing causality between RA characteristics or evolution and the presence of sarcopenia; (b) it exclusively involved elderly women, since it focused on the group most at risk for sarcopenia; and (c) it was localized to a single center, though it is believed to reflect typical characteristics of established RA patients.

Moreover, the sample size was determined using the best available prevalence at the time the study was planned, which was 4.5% for sarcopenia in individuals with RA (12). However, the study found an observed prevalence of 16.4%, significantly exceeding expectations. This significant discrepancy suggests that the sample size may have been too small to achieve optimal statistical power and precision, considering the actual variability within the study population.

Considering all these limitations, caution must be exercised in generalizing findings. Indeed, broader, longitudinal studies are needed to confirm these observations and extend them to a more diverse population. Future studies should also consider recalculating the necessary sample size using this newly observed prevalence to enhance the robustness of the findings.

Nevertheless, it makes significant contributions to the field by comparing EWGSOP-1 and EWGSOP-2 criteria for screening sarcopenia in an elderly female RA cohort, thus constituting a novel approach in this area. Additionally, it evaluates the SARC-F questionnaire's effectiveness in detecting sarcopenia among RA patients, an aspect not previously explored.

## Conclusion

This study, conducted among elderly Spanish women with RA, highlights that the detected prevalence rates of sarcopenia significantly differ based on the application of EWGSOP1 or EWGSOP2 criteria. Moreover, the SARC-F questionnaire shows good effectivity for predicting sarcopenia following the EWGSOP2 criteria, currently the most widely accepted in clinical practice. This underscores the importance of selecting appropriate diagnostic strategies for sarcopenia in RA patients, which can impact both clinical outcomes and management strategies.

## Data availability statement

The raw data supporting the conclusions of this article will be made available by the authors, without undue reservation.

## Ethics statement

The studies involving humans were approved by the IDIBELL Clinical Research Ethics Committee of Bellvitge. The studies were conducted in accordance with the local legislation and institutional requirements. The participants provided their written informed consent to participate in this study.

## Author contributions

LV-M: Data curation, Formal analysis, Investigation, Methodology, Validation, Writing – original draft, Writing – review & editing. CG-V: Conceptualization, Data curation, Formal analysis, Investigation, Methodology, Software, Validation, Writing – original draft, Writing – review & editing. MM: Data curation, Investigation, Methodology, Software, Writing – original draft, Writing – review & editing. LB-A: Data curation, Investigation, Methodology, Software, Writing – original draft, Writing – review & editing. DB: Data curation, Formal analysis, Investigation, Methodology, Software, Writing – original draft, Writing – review & editing. JN: Data curation, Investigation, Methodology, Software, Writing – original draft, Writing – review & editing. XJ: Data curation, Investigation, Methodology, Software, Writing – original draft, Writing – review & editing. JMN: Conceptualization, Formal analysis, Investigation, Methodology, Project administration, Supervision, Validation, Writing – original draft, Writing – review & editing.
